# Conditional Artificial Potential Field-Based Autonomous Vehicle Safety Control with Interference of Lane Changing in Mixed Traffic Scenario

**DOI:** 10.3390/s19194199

**Published:** 2019-09-27

**Authors:** Kai Gao, Di Yan, Fan Yang, Jin Xie, Li Liu, Ronghua Du, Naixue Xiong

**Affiliations:** 1 College of Automotive and Mechanical Engineering, Changsha University of Science & Technology, Changsha 410114, China; kai_g@csust.edu.cn (K.G.); yandi@stu.csust.edu.cn (D.Y.); csust.xiejin@gmail.com (J.X.); lukeliuli@csust.edu.cn (L.L.); csdrh@csust.edu.cn (R.D.); 2 Hunan Key Laboratory of Smart Roadway and Cooperative Vehicle-Infrastructure Systems, Changsha 410114, China; 3 School of Information and Security Engineering, Zhongnan University of Economics and law, Wuhan 430073, China; 4 College of Intelligence and Computing, Tianjin University, Tianjin 300350, China; xiongnaixue@gmail.com

**Keywords:** mixed traffic, lane change, autonomous vehicle safety, SVM, C-APF, car-following

## Abstract

Car-following is an essential trajectory control strategy for the autonomous vehicle, which not only improves traffic efficiency, but also reduces fuel consumption and emissions. However, the prediction of lane change intentions in adjacent lanes is problematic, and will significantly affect the car-following control of the autonomous vehicle, especially when the vehicle changing lanes is only a connected unintelligent vehicle without expensive and accurate sensors. Autonomous vehicles suffer from adjacent vehicles’ abrupt lane changes, which may reduce ride comfort and increase energy consumption, and even lead to a collision. A machine learning-based lane change intention prediction and real time autonomous vehicle controller is proposed to respond to this problem. First, an interval-based support vector machine is designed to predict the vehicles’ lane change intention utilizing limited low-level vehicle status through vehicle-to-vehicle communication. Then, a conditional artificial potential field method is used to design the car-following controller by incorporating the lane-change intentions of the vehicle. Experimental results reveal that the proposed method can estimate a vehicle’s lane change intention more accurately. The autonomous vehicle avoids collisions with a lane-changing connected unintelligent vehicle with reliable safety and favorable dynamic performance.

## 1. Introduction

Autonomous driving has been a focus of industry and academia in recent decades, with pledges of safety, convenience, and energy efficiency. The challenges of the autonomous vehicle include the unknown intentions of other road users; communication between vehicles and with the road infrastructure is a possible approach to enhance awareness and enable cooperation [1]. Significant research on autonomous vehicles is underway, not only in the U.S. and China, but also in Europe and other parts of the world. According to some in the industry, it is only a matter of time before these kinds of advances allow people to outsource their daily commute to a computer on an autonomous vehicle. However, traditional human-controlled vehicles will be dominant for an extended period in urban transportation networks [2]. Such vehicles—namely, connected unintelligent vehicles (CUVs)—may be equipped with communication and Global Positioning System (GPS) devices for common application by government mandate, but without additional expensive sensors. Although the sensing ability of an autonomous vehicle is potent, the CUV’s information is limited [3]. Only the low-level necessary safety information, including speed and position, is available by vehicle-to-vehicle (V2V) communication. The motion decisions of autonomous vehicles suffer from the difficulty of predicting the drive intentions of CUVs, which leads to traffic accidents involving vehicles such as Uber and Google cars. It is a significant challenge for the autonomous vehicle’s control decisions when travelling in a mixed traffic scenario with autonomous vehicles and CUVs [4].

One of the danger scenarios is the lane change behavior of adjacent CUVs when the autonomous vehicle is travelling in car-following mode [5]. The CUV driver’s lane change intention is difficult to predict, and may lead to a collision with an autonomous vehicle travelling in an adjacent lane. According to National Highway Traffic Safety Administration (NHTSA) research data, traffic accidents caused by lane changes accounted for 27% of all accidents, and traffic accidents caused by driver factors accounted for 93%. At the same time, the statistics of China field operational tests (China-FOT) also showed that dangerous accidents resulting from lane changes accounted for 23.91% of total accidents [6]. Even if the lane change behavior of a CUV does not lead to a collision, the autonomous vehicle travelling in car-following mode has to regulate its speed to maintain a safe distance, and may be required to perform emergency braking [7]. Because the distance may be short, lane change behavior could be dangerous. Also, repeated deceleration and acceleration of an autonomous vehicle increases fuel consumption, and significantly affects ride comfort because sudden lane changes shorten the response time for autonomous vehicles. Therefore, it is crucial to predict this kind of lane change behavior in advance so that the control decision of autonomous vehicles can also be made in advance [8].

In this paper, a learning-based algorithm is developed to predict the CUV’s lane change intention, and a car-following controller is designed by incorporating the lane-change intentions of the CUV. The main contributions of this manuscript are as follows: (1) A safety control issue for an autonomous vehicle in a mixed traffic scenario with unintelligent vehicle lane-change interference is investigated. This is likely to remain a typical scenario in the future, and a number of studies have focused on it. (2) A kinematic information-based lane-change intention prediction model is constructed for the unintelligent vehicle with a given accuracy, which predicts the vehicle’s lane change intention with a 2 s prediction horizon. The proposed method does not require expensive sensors mounted on the vehicle as in other methods. (3) A real-time control strategy considering lane-change interference with low computational complexity for autonomous vehicle’s longitudinal safety is proposed.

The rest of this paper is organized as follows: Section 2 discusses the state of the art in related work. Section 3 describes the applicable scenario and primary conditions of the algorithm proposed in the manuscript. Section 4 discusses the proposed lane change intention prediction algorithm and I-APF-based autonomous vehicle’s controller. Section 5 presents the simulation result to validate the algorithm performance.

## 2. Related Work

The prediction of vehicle lane change behavior is mainly divided into kinematic information-based and driver behavior-based methods. Kinematic information is directly related to vehicle lane-changing. Some information, including speed, acceleration, and position during lane-changing, is easy to access, and is utilized to predict the lane change intention. Puneet Kumar et al. [9] proposed an online lane change intention prediction method. This method uses the multi-class probability output of a support vector machine as the input of a Bayesian filter. The output of the Bayesian filter is utilized for the final prediction of lane changes. This method uses the vehicle’s lateral position, steering wheel angle, vehicle lateral speed, and steering wheel angular velocity as input information of the prediction system. Real-time data acquisition is performed by a lane tracker installed on the vehicle, and data is collected for model training and testing. Ranjeet Singh Tomar et al. [10] designed an artificial neural network (ANN) to predict the trajectory of the lane change. First, the historical trajectory of the lane-changing vehicle and nearby vehicles was used to predict the short-term and long-term trajectory of the vehicle. Ma Guocheng et al. [11] extracted seven motion properties of vehicles travelling in main lanes and adjacent lanes. Information that cannot be directly obtained by sensors was estimated by a Kalman filter. Then, a fuzzy support vector machine was designed to establish a lane change intention recognizer. These above-mentioned methods are heavily based on kinematic information to predict lane change intentions. However, some of the information cannot be measured for a traditional vehicle, such as the steering wheel angle, which is not available for the hydraulic power steering system in a traditional vehicle. To add a V2V onboard electronic control unit (ECU) is more applicable, while mounting angle sensors may not be acceptable for many vehicle owners.

What’s more, some researchers pay attention to driver behavior detection method, due to the fast development of computer and image recognition technology [12]. Unlike vehicle kinematic data, driver behavior, such as head and eye movements, can give an early clue about the driver’s intention [5]. Lemmer et al. used a driving simulator to assess the timing and quantity of data needed to predict driving intentions [13]. They concluded that a 10-second eye-gazing information window best predicts intent, and a 5-second eye-gazing information window performs best because of less noise. Young-Min Jang et al. proposed a lane-change intention prediction model, based on the change in the pupil size of the human eye, suitable for an intelligent driving assistance system [14]. Intention-oriented eye tracking can be viewed as a cognitive process of information gathering, which provides an early indication of driver mental states. Most often, a driver will shift their eye gaze on purpose, which makes eye movement an essential signal for the prediction of lane change intentions [15]. A significant challenge to gathering information from the eye is the task of eye tracking. It is not easy to accurately detect the eye and track the pupil due to the physical characteristics of the eye (small scale, occlusion, etc.). Similarly, head motion was regarded as the most critical factor for intention prediction [16]. When an outside stimulus occurs and the driver is faced with a non-goal-oriented task, they will rotate their head [15]. Although it was found that head tracking is most relevant to the prediction of driver intentions, head tracking is hard to realize for commercial applications. On the whole, although image-based driving behavior detection has precise accuracy, it consumes many computing resources and is greatly affected by external influences. This type of method is not suitable for a mixed traffic scenario of CUVs and autonomous vehicles.

Therefore, a kind of cooperative lane change for connected vehicles attracts the attention of many scholars. Architecture and protocol of cooperative lane change for connected autonomous vehicles (CAVs) is proposed in [17]. In addition, the authors proposed a system that could accurately convey the lane change intention through V2V communication, instead of estimation using sensors and statistical models. A deep reinforcement learning method is utilized in [18]. It reveals that the reward of the system should consider the overall traffic efficiency instead of the travel efficiency of an individual vehicle. A decentralized cooperative lane-changing decision-making framework for connected autonomous vehicles is proposed in [19], which is composed of three modules, i.e., state prediction, candidate decision generation, and coordination. A cooperative lane-changing strategy using a transferable utility games framework is proposed in [20]. Gaps in traffic are created in exchange for monetary compensation to make vehicles to engage in transactions. The proposed utility transfer allows vehicles engaged in such transactions to achieve Pareto efficient payoffs, which will consume substantial computing resources. All of the above methods show good security and efficiency. However, they are based on CAVs. In mixed traffic scenarios, CUVs and autonomous vehicles cannot form a cooperative mode, and CUV drivers are reluctant to form a collaboration in many cases. Therefore, it is necessary for the autonomous vehicle to be always vigilant against the CUV traveling in the adjacent lane, and to estimate in advance whether it has a lane change intention in an un-cooperative mode.

Since it is difficult to form a cooperative mode lane change for CUV, the sensors’ data on board becomes significant to predict the lane change intention. Therefore, data fusion technology based methods have emerged to predict the lane change intention due to the development of sensor network technology [21,22]. A novel preprocessing algorithm for the ADAS is proposed to improve the accuracy of classifying the driver’s intention to change lane by augmenting necessary measurements from conventional onboard sensors [23]. The effects of reset control on the alleviation of the rise time, settling time, and overshoot limitations are explored for a lane change maneuver under a set of demanding design conditions to guarantee a suitable ride quality and a swift response [24]. A two-stage data-driven approach is proposed to classify driving patterns of surrounding vehicles, using Gaussian mixture models (GMM) [25]. Vehicles’ short-term lateral motions are predicted based on real-world vehicle mobility data, where several critical kinetic features and higher-order kinematic variables are utilized. A unified framework for surrounding vehicle maneuver recognition and motion prediction using vehicle-mounted perceptual sensors is proposed, which is based on an understanding of motion patterns of freeway traffic and the effect of inter-vehicle interactions. It reveals that incorporating a model that takes into account interactions between surrounding vehicles for simultaneously predicting each of their motions leads to better prediction as compared to predicting each vehicle’s motion independently [26]. All of these methods are based on sensor data on lane-changing vehicles, and encouraged by network optimization for reducing latency [27,28], which may be more useful for mounting these sensors on vehicles. However, more sensors are required to realize these methods.

In order to improve traffic efficiency and ensure the safe driving of autonomous vehicles, many control strategies based on optimization and intelligent algorithms have emerged [29,30]. A right-of-way assignment strategy by setting clear collision avoidance conditions to improve the lane-changing model of responsibility-sensitive safety (RSS) is proposed in [31]. Negotiation is used to improve the utilization of limited road resources, and numerical testing results reveal that the process of right-of-way assignment is more efficient, reasonable, and safe. A quantitative collision risk assessment algorithm is studied in [32], where a set of local path candidates is produced by the lane-based probabilistic motion prediction of surrounding vehicles. Sufficient simulation data are transferred by transfer learning methods to supplement the training set with few labeled real data, thus improving the performance of behavior recognition in the real world [33]. Analysis of the data for lane change intent is performed using a sparse Bayesian learning methodology. Lane position, vehicle parameters, and driver head motion are utilized in this method [34]. Several kinds of machine learning are used in [35] to predict a lane change maneuver. Features relating to the position and the speed of the target vehicle with respect to the host vehicle are extracted to train and test the machine learning. A real-time LSTM-based algorithm is proposed to predict trajectories for heterogeneous traffic-agents in an urban environment in [36]. There is no assumption about traffic conditions or the number of agents. These methods are based on a large number of samples and expensive lidar or cameras, which are not applicable for most vehicles.

In order to cope with mixed traffic scenarios, it is necessary to install some low-cost, easy-to-operate equipment on traditional vehicles. A set of low-cost Global Positioning System/inertial measurement unit (GPS/IMU) sensors and an odometry captor for collecting velocity measurements is utilized and mounted on the vehicle to predict lane changes in highways. Extended Kalman filters (EKFs) running in parallel and integrated by an IMM-based algorithm provide positioning and maneuver predictions to the user [37]. Based on this method, a cooperative collision avoidance system has been developed incorporating digital maps [38]. For many unintelligent vehicles, GPS and vehicle to everything (V2X) may be equipped, but IMU sensors are not available due to their high cost. Furthermore, for human-centered driving, forming a cooperative with an autonomous vehicle is difficult. The characteristics of the available data should be fully utilized to predict lane change intentions. 

## 3. Lane Change Behavior Model

### 3.1. Applicable Scenario and Basic Conditions

This paper studies the estimation of CUV lane change intentions and the autonomous vehicle safety control strategy in a common and high-risk mixed traffic scenario. The CUV cuts into the lanes of adjacent autonomous vehicles in a non-cooperative manner. This scenario is expected to be the most ubiquitous in the near future. The CUV and autonomous vehicle are travelling in adjacent lanes. To use the method proposed in this manuscript, the following conditions should be met:

(1) The CUV must be equipped with GPS and wireless transmission equipment, which can be conveniently installed;
(2) The speed, acceleration, and ID of the CUV can be periodically sent to autonomous vehicles by V2X;
(3) The autonomous vehicles are travelling freely or in a car-following mode;
(4) The CUV is human driver oriented, and there is not a cooperative manner between the CUV and autonomous vehicles.

The first condition is relatively common because of navigation requirements and government mandates. The speed and acceleration can be obtained via GPS or vehicle bus, and then integrated with onboard V2X equipment to be broadcast to surrounding vehicles. On urban roads and highways, autonomous vehicles usually travel in car-following mode, which is a safe and energy-saving driving mode according to research results. However, a human driver-oriented CUV travelling in the adjacent lane is a significant safety risk to autonomous vehicles, because the very short lateral distance can easily lead to collisions when the CUV unpredictably changes lanes in a non-cooperative manner. The sensors used in the proposed method are shown in Figure 6. The functions of the speed sensor, GPS, and V2X are described in the above text. A radar mounted on the autonomous vehicle is used to detect the relative distance once the CUV’s lane change prediction is made.

It is well known that vehicles traveling in adjacent lanes are dynamically changing. Two cars that are traveling side-by-side are defined as a couple, denoted by the symbol Co<Ui, Ai>, where Ui denotes the CUV and Ai denotes an autonomous vehicle. When the lateral positions of Ui and Ai are within a specific range, two cars that may collide due to a lane change are defined as a couple. From the actual situation of traffic, the couple frequently changes for the same autonomous vehicle.

### 3.2. Analysis of Lane-Changing Behavior 

**Figure 1:**
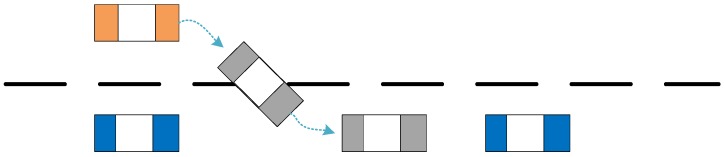
Schematic diagram of lane change behavior in adjacent lanes.

Figure 1 depicts the process of a CUV cutting into an adjacent lane. The time when the lane change vehicle crosses the lane line is defined as the change-making time. Usually, the driver will observe the surrounding environment before changing lanes, adjust the speed of the vehicle, and look for the timing of the lane change. Therefore, the speed before the lane change will fluctuate more than the lane-keeping. Compared with the lane-keeping mode, the vehicle will continuously move to one side when the lane is changed, so the lateral vehicle speed of the vehicle can also be used as a discriminating indicator for the prediction of lane change intention. A driver would like to change lane when his vehicle speed is higher than a preceding vehicle’s speed, and the relative distance between the driver and the front vehicle is shortened. Therefore, the distance to the front car may also be used as a discriminating indicator of whether the driver will change his lane.

**Figure 2:**
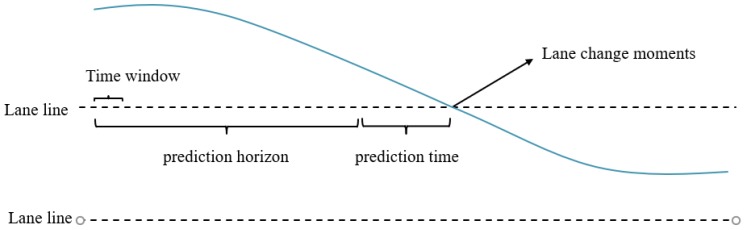
Schematic diagram of time sequence for lane change intention prediction.

Based on the above analysis, the longitudinal velocity and acceleration variation of the CUV over some period, the lateral velocity variation, and the headway interval can be used as indicators for the prediction of lane change intentions. Therefore, it is possible to predict whether the CUV will change lanes after several seconds using the data before the lane change moment. This data interval is denoted as the prediction horizon, which consists of several time windows. The CUV’s state data of each time window is utilized as a feature sample that can be used to train the vehicle lane change intention model. The relationship between these parameters is shown in Figure 2.

Remark 1: To predict the lane-changing behavior of CUV on the adjacent lane is of great challenge for the autonomous vehicle due to limited sensor information available on the CUV. Although the sensors are most important for lane-changing prediction of the CUV and safety control of the autonomous vehicle, only necessary and available sensors on the CUV and autonomous vehicle are considered in the proposed method. The longitudinal velocity and acceleration variation of the CUV are calculated by the information from speed sensors. The lateral velocity variation and the headway interval of the CUV are gained by the GPS. As soon as the lane-changing is predicted by the proposed method, the safety controller of the autonomous vehicle begins to work with the ranging sensor radar. 

Since the CUV generally does not have an ECU with strong processing and computing power, the autonomous vehicle has a powerful arithmetic processor. Therefore, when the autonomous vehicle receives information revealing that a CUV is travelling in an adjacent lane, the lane change behavior intention prediction of the CUV in the couple is performed by the autonomous vehicle. Usually, the duration that the couple remains the same will be short. Therefore, a machine learning method with a small number of samples, strong generalization ability, and enough prediction accuracy is needed. In the model adopted in this paper, an algorithm runs on the autonomous vehicle to predict the change intention of the Ui in the couple and, based on the predicted result, then decides the Ai’s motion control.

## 4. The Proposed Lane Change Intention Prediction and Safety Controller

**Figure 3:**
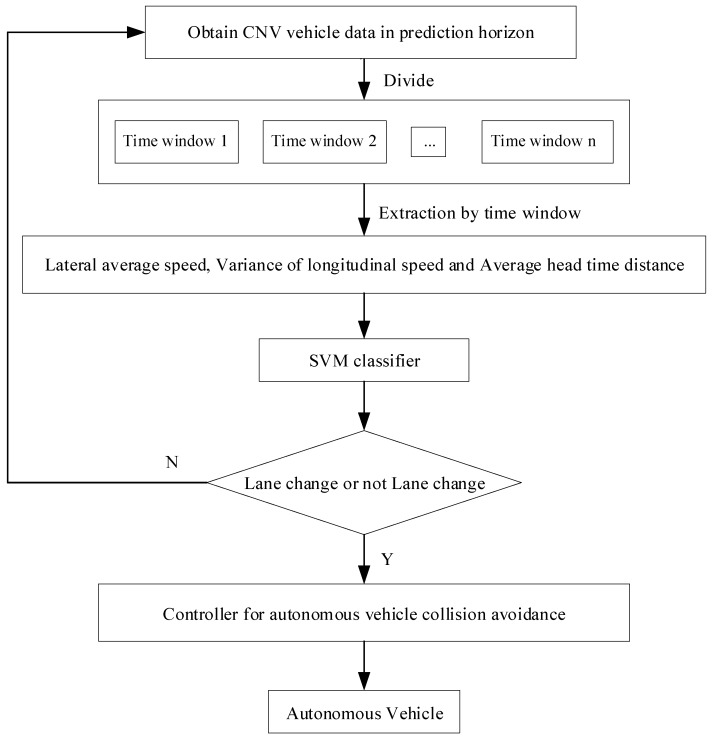
The overall diagram of lane change intention prediction and safety controller design.

The overall block diagram of the proposed lane change intention prediction and safety controller is shown in Figure 3. Firstly, vehicle data in the CUV’s prediction horizon are obtained, including longitudinal vehicle speed, lateral position, and headway time. Then these data are divided into N time windows according to a certain length of time. The lateral average speed, the variance of longitudinal speed, and the average headway time of CUV vehicles in each time window are calculated, and these data are used to judge whether the vehicles will change lanes or not. The support vector machine (SVM) classifier is used to decide whether the CUV vehicle will change lane. If the classifier judges that the CUV vehicle will change lane, it will start the controller for autonomous vehicle collision avoidance.

### 4.1. Interval-Based Support Vector Machine (I-SVM)

**Figure 4:**
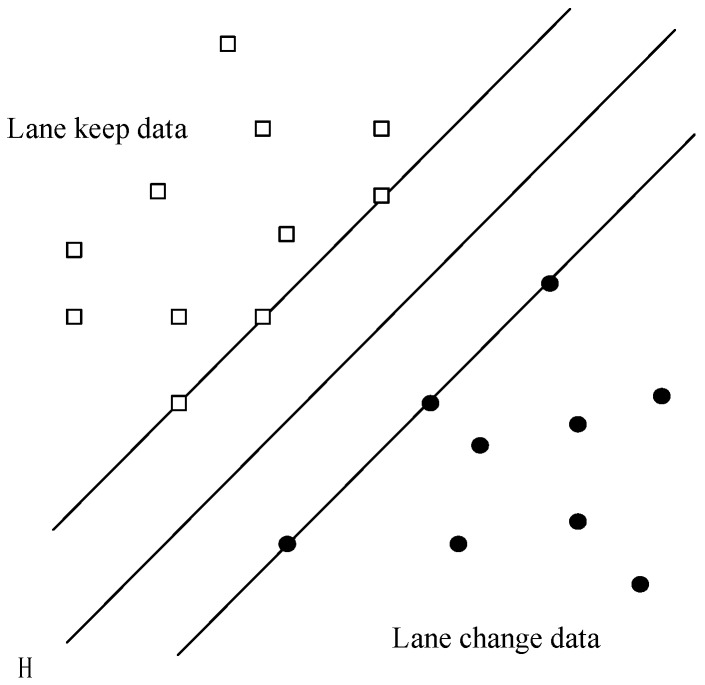
Schematic diagram of connected unintelligent vehicle (CUV) data classification.

A machine learning-based lane change intent prediction is proposed in this paper. As shown in Section 3.1, the speed, acceleration, GPS, and ID of the CUV can be sent to the autonomous vehicle by V2X. Then, the autonomous vehicle can store the information and develop an algorithm to predict the lane change intention. The CUV travelling in the adjacent lane changes frequently, so this algorithm is used for a few samples. The feature requires a small sample and strong generalization ability. As everyone knows, the SVM is suitable for use in this paper. If the data is linearly separable, a linear function can be found to separate the samples. When the spatial dimension is not considered, such a linear function is called a hyperplane. As shown in Figure 4, H is the optimal hyperplane, which can be used to separate data. Types are separate and can maximize the classification interval.

If the data are linearly inseparable, the hyperplane shown in Figure 4 will not be found. kernel functions will be used to map low-dimensional linear indivisible problems to high-dimensional ones to become linear separable problems. In this paper, the radial basis function (RBF) is chosen as the kernel function of SVM. The radial basis function can realize non-linear mapping, as expressed in Equation (1):

(1) $$k\left(x,x_{c}\right)=e^{-\frac{{\left|\left|x-x_{c}\right|\right|}^{2}}{2\sigma^{2}}}$$

Equation (1) is a Gauss kernel function, which is the most common radial basis function. In the equation, *x_c_* represents the center of the kernel function, *σ* is the width parameter of the function and represents the radial range of the function.

The start of the CUV lane change intention prediction program is given as Algorithm 1.

**Algorithm 1:** Algorithm for lane change prediction of the CUV **Input:***v_i_*, longitudinal speed of the CUV that was acquired for the *i* th time in a time window; *x_b_*, lateral position of the CUV at the beginning of a time window; *x_e_*, lateral position of the CUV at the ending of a time window; *n*, number of data acquisitions in a time window; *h_i_*, headway time of the CUV that was acquired for the *i* th time in a time window; *t*, duration of a time windowOutput:1: c, predicted result.2: **Algorithm begin:**3: $v_{al}=\frac{\sum_{1}^{n}{\left(v_{i}-{\bar{v}}_{l}\right)}^{2}}{n-1}\;$ 4: where ${\bar{v}}_{l}=\frac{s}{t}\;$ 5: ${\bar{v}}_{a}=\frac{\left|x_{e}-x_{b}\right|}{t}\;$ 6: $h_{a}=\frac{\sum_{1}^{n}h_{i}}{n}\;$ 7: Combining $v_{al}$, ${\bar{v}}_{a}$, $h_{a}$ from *n* time windows into feature vectors 8: Enter the feature vector into the SVM classifier 9: **if** SVM classifier determines that CUV will change lanes **then** 10: **c** = 1 11: else 12: c = −1 13: return c14: **Algorithm end**

### 4.2. Data Sample and Pre-Process

**Figure 5:**
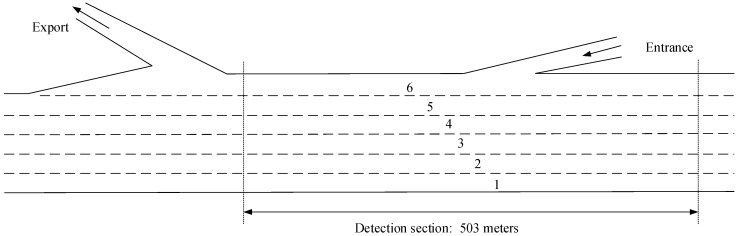
I-80 road structure.

The vehicle trajectory data of the I-80 road section in this paper were selected from the public data set NGSIM [39] as the data source. Sample data used to train the lane change prediction model were extracted from the public data set NGSIM. The NGSIM project is funded by the US Federal Highway Administration, and these trajectory data provide a valuable basis for research on driving behavior. The vehicle trajectory data provided by the data set includes: vehicle speed, acceleration, lateral position, relative distance to the preceding vehicle, and the headway distance. The sampling frequency is 10 Hz, and the road structure of the I-80 road section is shown in Figure 5.

Because the vehicle’s longitudinal speed, lateral speed, and headway distance can reflect the driver’s intention to change lanes, this paper selects the variance of the vehicle’s longitudinal speed, the lateral speed, and the headway of the preceding vehicle to determine whether the driver will change lanes.

Remark 2: There are other states related to the lane change behavior of vehicles. For example, drivers usually employ the vehicle turn signal when they make a turn. However, there exists a random element for different drivers, and the turning time and the light change time may be extremely different for the same driver. The lane change intention of an unintelligent vehicle is anticipated predicting, so the use of the turn signal is not suitable to be sent to autonomous vehicles to predict the lane change.

The steps for extraction are as follows:

(1) Find the lane change vehicle and remove the 7th lane change vehicle, because the 7th lane is the gateway and will affect the judgment result.

(2) Take the vehicle data for the first 20 s of the lane change, and 200 sets of data for another car.

(3) Calculate the longitudinal speed variance, average lateral speed and average head time of the vehicle as a time window every second.

(4) The vehicle longitudinal vehicle speed variance, the average lateral vehicle speed, and the average headway time interval in each time window are combined to form a feature input of the support vector machine.

This paper extracts 100 lane change vehicle data sets and 101 lane keeping vehicle production training sets and test sets, taking 160 vehicle data for training and 41 vehicles for testing. In order to ensure the classification performance of the classifier, the data needs to be normalized to [−1, 1].

### 4.3. Control Design for Autonomous Vehicle Collision Avoidance 

A. Artificial Potential Field

The artificial potential field (APF) method is a real-time obstacle avoidance method for manipulators and robots based on the concept of the artificial potential field proposed by Khatib. The basic idea is to abstract the working environment of the robot into a virtual force field space to construct an artificial potential field, which acts together with the attraction field of the target and the repulsive field of the obstacle, and to plan the motion path of the robot under the action of the force field.

$$\mathrm{Attraction}\;\mathrm{Field}:\;U_{att}\left(X\right)=\frac{1}{2}k{\left(X-X_{d}\right)}^{2}\;$$

$$\mathrm{Attraction}:\;F_{att}=-\nabla U_{att}\left(X\right)=-k\left(X-X_{d}\right)\;$$

where *k* is the attraction gain coefficient, *X* is the position vector of the vehicle, *X_d_* is the position vector of the target point, and attraction is the negative gradient of the attraction field.

$$\mathrm{Repulsive}\;\mathrm{Field}:\;U_{rep}\left(X\right)=\{\begin{array}{ll} \frac{1}{2}\eta {\left(\frac{1}{\rho }-\frac{1}{\rho_{0}}\right)}^{2}, & if\;\rho \leq \rho_{0}\\ 0, & if\;\rho >\rho_{0}\; \end{array}$$

$$\mathrm{Repulsive}:\;F_{rep}=-\nabla U_{rep}\left(X\right)=\{\begin{array}{ll} \eta \left(\frac{1}{\rho }-\frac{1}{\rho_{0}}\right)\frac{1}{\rho^{2}}, & if\;\rho \leq \rho_{0}\\ 0, & if\;\rho >\rho_{0} \end{array}\;$$

where *η* is the repulsive gain coefficient, *ρ* is the distance between the robot and the obstacle, and *ρ*_0_ is the repulsive influence range, which will not be affected by the repulsive field outside the repulsive influence range. 

B. Design of Vehicle Conditional Artificial Potential Field

**Figure 6:**
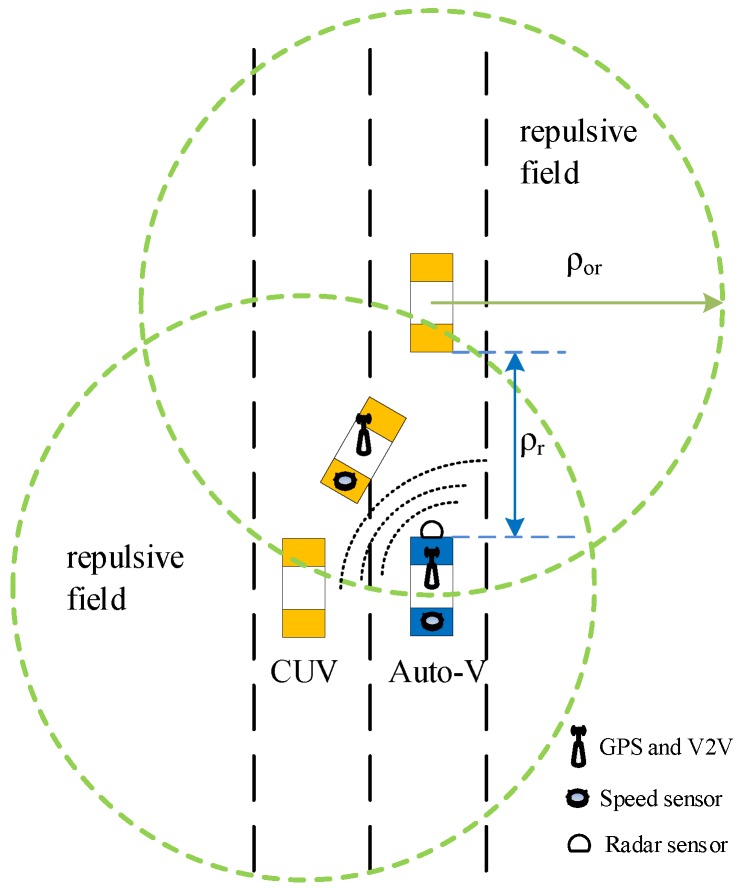
A repulsive field centered on a lane change vehicle.

The artificial potential field is highly suitable for a vehicle collision avoidance system. A conditional artificial potential field (C-APF) is proposed for vehicle lane-changing safety control. The C-APF is different from the traditional APF. It will form only when conditions are satisfied, namely, that the lane change prediction system predicts that a CUV will change lanes. In the C-APF, the direction of repulsion and attraction is only longitudinal, that is, in the lane direction. As shown in Figure 6, the autonomous vehicle is running at a constant speed. At this time, the lane change prediction system predicts that vehicles in adjacent lanes will change lanes, and the vehicle collision avoidance system will form a repulsive field centered on the CUV. The closer the distance between the autonomous vehicle and the CUV vehicle, the more repulsive the autonomous vehicle will be, so that it will have greater negative acceleration and achieve rapid deceleration to ensure safety. When the distance between the autonomous vehicle and the CUV is greater, it will suffer less repulsion and slow down less to ensure comfort. The influence range of the repulsion field is *ρ_or_*. The size of *ρ_or_* is related to the speed of the autonomous vehicle, and the relationship is expressed by Equation (2).

(2) $$\rho_{or}=l=d_{0}+hv_{t}$$

where *l* represents the safe distance while driving, and *d*_0_ represents the minimum safe distance, i.e., the minimum distance when the front car suddenly stops, and no collision occurs. *h* is the time headway, and *v_t_* represents the speed of the autonomous vehicle. *ρ_or_* represents the distance between the autonomous vehicle and the CUV. When *ρ_r_* is less than *ρ_or_*, the autonomous vehicle will be affected by the repulsion field, which is expressed by Equations (3) and (4).

(3) $$U_{rep}=\frac{1}{2}\eta {\left(\frac{1}{\rho_{r}}-\frac{1}{\left(d_{0}+hv_{t}\right)}\right)}^{2}$$

(4) $$F_{rep}=-\nabla U_{rep}=\eta \left(\frac{1}{\rho_{r}}-\frac{1}{\left(d_{0}+hv_{t}\right)}\right)\frac{1}{\rho_{r}{}^{2}}$$

*η* is the repulsion gain coefficient. The larger *η*, the greater the repulsion exerted on the autonomous vehicle at the same distance. The autonomous vehicle will decelerate faster, which means that the obstacle avoidance strategy is more conservative.

**Figure 7:**
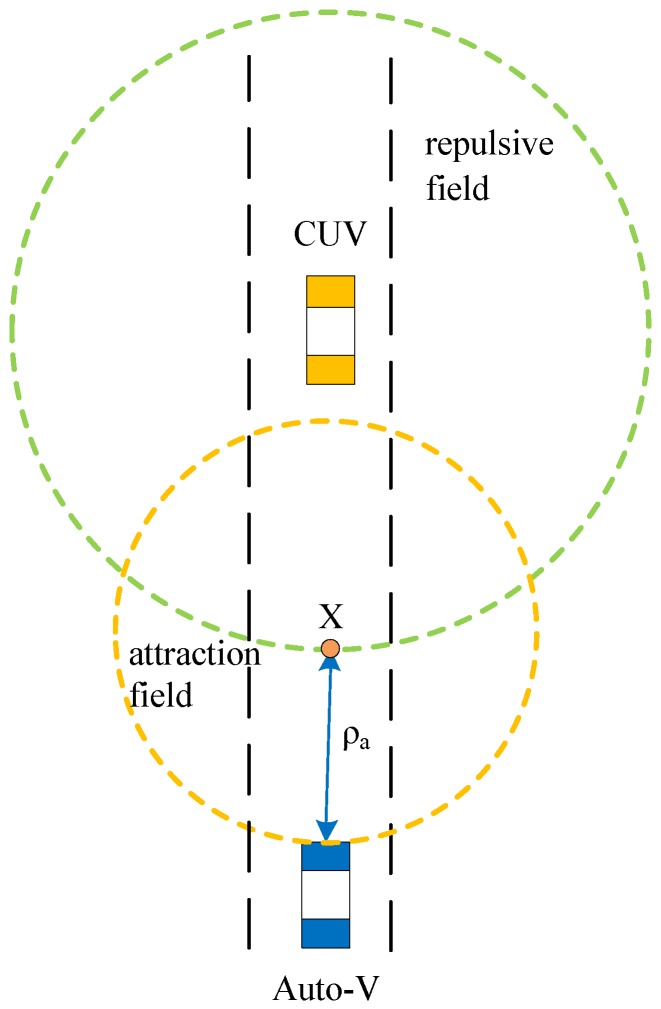
Autonomous vehicle affected by attraction field.

When *ρ_r_* is larger than *ρ_or_*, the autonomous vehicle is no longer affected by the repulsion field, but by the attraction field. As shown in Figure 7, the center of the attraction field is the *X* point; the attraction field and attraction are expressed by Equations (5) and (6).

(5) $$U_{att}\left(X\right)=\frac{1}{2}k\rho_{a}{}^{2}$$

(6) $$F_{att}=\nabla U_{att}\left(X\right)=k\rho_{a}$$

where *k* is the attraction gain coefficient, and *ρ_a_* is the distance from the autonomous vehicle to point *X*. The larger *ρ_a_*, the greater the attraction. The attraction will make the autonomous vehicle achieve positive acceleration, thus accelerating the vehicle. When the speed of the autonomous vehicle is equal to the speed of the CUV, the autonomous vehicle will no longer be affected by the attraction field and will maintain a uniform speed to follow the CUV.

The control algorithm for the autonomous vehicle to avoid colliding with a CUV is shown in Algorithm 2. When the CUV is travelling in the adjacent lane of an autonomous vehicle and drives into the safety-sensitive area of the autonomous vehicle, it is denoted as Co<Ui, Ai>. As Co<Ui, Ai> is formed, the autonomous vehicle receives information periodically, stores these messages in memory, and starts the CUV lane change intention prediction program as shown in Algorithm 2.

**Algorithm 2:** Control Algorithm for Autonomous Vehicle to avoid Collide with CUV**Input:**, *ρ_r_*, the distance between the autonomous vehicle and the CUV; *ρ_or_*, the influence range of repulsion field; *ρ_a_*, the distance between automatic driving vehicle and target point; m, the quality of autonomous vehicle; *k*, the attraction gain coefficient; *η*, the gain coefficient; A, the variance of longitudinal vehicle speed of CUV in a time window; *V_La_*, average lateral velocity of CUV vehicle in a time window; *h*, average head time distance of CUV in a time window; *v*_1_, the current speed of autonomous vehicle; *v*_2_, the current speed of CUV;Output:1: a, the acceleration of self-driving vehicle.2: **Algorithm begin:**3: Start controller for autonomous vehicle collision avoidance4: **if**
*ρ_r_* < *ρ_or_*
**then**5: *a* = *a*_1_ 6: where $a_{1}=-\frac{\eta \left(\frac{1}{\rho }-\frac{1}{\rho_{0}}\right)\frac{1}{\rho^{2}}}{m}\;$ 7: **else if**
*ρ_r_* > *ρ_or_*
**then** 8: *a* = *a*_2_ 9: where $a_{2}=\frac{k\rho_{a}}{m}\;$ 10: **if**
*v*_1_ = *v*_2_
**then**11: Close controller for autonomous vehicle collision avoidance 12: return *a* 13: **Algorithm end**

## 5. Simulation and Result Analysis

### 5.1. Validating of Driver’s Lane-Changing Behavior Intention

**Figure 8:**
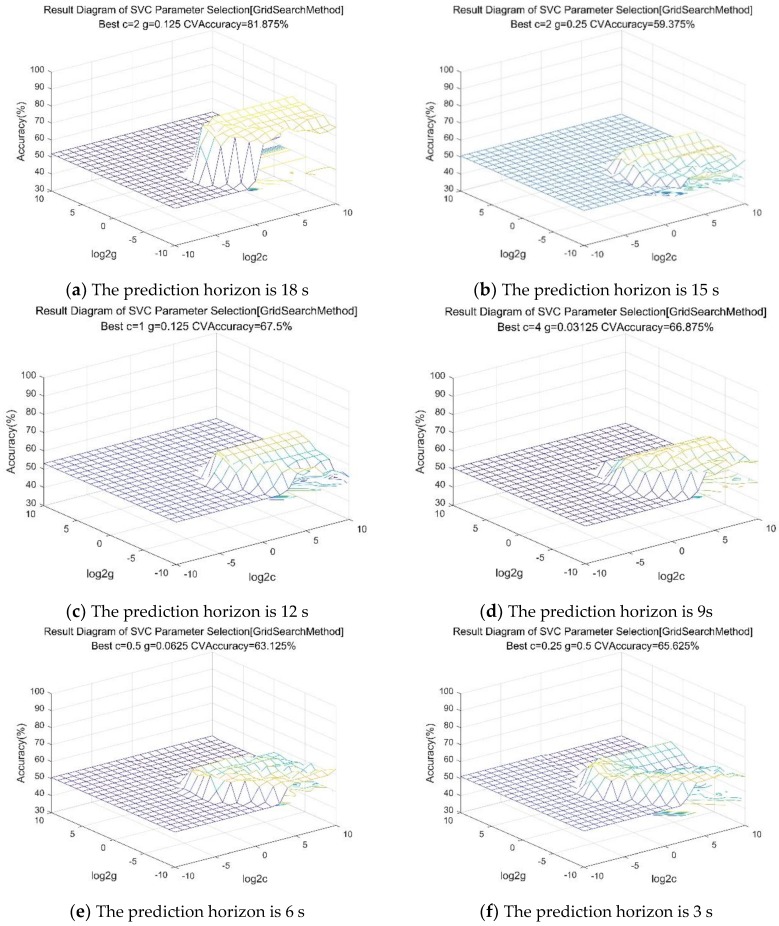
Parameter optimization results of I-SVM.

The libsvm toolbox in the MATLAB environment is used to model the driver’s lane-changing behavior, and select the radial basis function as the kernel function of the SVM classifier. Firstly, the processed training set data and test set data is labeled. The lane change markers are 1 and −1. The model parameters c and g should be determined before training. c is a penalty coefficient, representing tolerance of errors. The larger c, the more intolerable errors appear. g implicitly determines the distribution of data after mapping to a new feature space. In this paper, the grid method is used to optimize the parameters of c and g, and the optimal combination of parameters is obtained. Finally, the optimal c and g are imported into the model for training. In this paper, the length of the time window is 1 s, and the prediction horizons are 18 s, 15 s, 12 s, 9 s, 6 s and 3 s, respectively. The data sets for different prediction horizons are optimized by the grid method, and then the optimal parameter import model is used to start training. Finally, the optimal prediction horizon is selected as the predictive time domain of the classifier by analyzing the test results. The results of the optimization of different prediction horizon parameters are shown in Figure 8a–f.

The prediction accuracy of the training set and test set for different prediction horizons is shown in Table 1. Through cross-validation and prediction accuracy, it shows that the prediction accuracy of the prediction time domain is 18 s, and the prediction accuracy of the test set is 83.333%.

To validate the performance of the proposed I-SVM for lane-change prediction, BP neural networks and decision trees are used to predict the CUV’s lane change behavior on the same sample data. BP neural networks are often used to solve regression and classification problems. Lane change and no lane change are classification problems, so BP neural network can be used to predict lane change. A BP neural network is a feedforward neural network with error reverse propagation. Errors are reduced during training by continually modifying the weights between neurons. The different data of prediction horizons are chosen as inputs to the BP neural network to predict CUV lane change. The number of neurons in the hidden layer is obtained by empirical formula. The output layer has only one neuron. The output value ranges from [0, 1]. The output value is used to compare with 0.5. If it is greater than 0.5, it is a lane change. If it is less than 0.5, the lane is not changed. The prediction results are shown in Table 2.

It can be seen from Table 2 that the vehicle tracking data of 12 s before the CUV lane change is used for the lane change prediction, and the prediction accuracy is high, although only 57.64%.

The decision tree is also one of the classification algorithms, consisting of root nodes, non-leaf nodes, leaf nodes, and branches. The decision tree is used to make the lane change prediction. Data from different prediction horizons is chosen as feature vectors to build a decision tree, and the constructed decision tree is then used to perform the lane change prediction. The same test data is utilized to validate the prediction accuracy. The prediction results are shown in Table 3.

As can be seen from Table 3, the prediction accuracy of the decision tree algorithm is higher than that of the BP neural network. The prediction accuracy is highest when the prediction horizon is 9 s, and the prediction accuracy reaches 62.76%. From the above results, it can be seen that the BP and decision tree perform worse than the proposed I-SVM when used to predict the lane change intention from the same data set. In the following section, these algorithms are incorporated with an autonomous vehicle controller to validate the performance in a mixed traffic scenario.

### 5.2. Validating of C-APF Controller

MATLAB is used as a simulation platform to test the C-APF controller incorporated with the I-SVM, BP, and decision tree lane-change prediction algorithm. To validate the prediction, a no lane-changing prediction is also included in the test. Thus, four types of lane-changing prediction of C-APF controllers are tested in the same scenario, and the performance is compared in terms of the details of distance, speed, acceleration, energy consumption, and jerk. 

The test scenario is set as follows: the autonomous vehicle runs at a longitudinal speed of 40.32 km/h, and the CUV runs in the lane to the left of the autonomous vehicle with a speed of 39.6 km/h and lateral speed of 0.61 m/s to move into the autonomous vehicle’s lane. The CUV travels in front of the autonomous vehicle and the relative spacing is 7 m. The CUV changes lanes at the 17th second.

Remark 3: To match the test scenario to the autonomous concept test, many autonomous driving test cases and specifications have been researched in USA, Europe, China, etc. However, an international specification has not been drafted. Thus, a lane change test scenario from “A Framework for Automated Driving System Testable Cases and Scenarios” of NHTSA (2018) is selected. For lane change tests, the autonomous vehicle’s speed was set to 40.32 km/h (low speed scenario).

To calculate energy consumption during the test, the parameters related to the autonomous vehicle of this simulation are shown in Table 4.

A. Speed of the autonomous vehicle

**Figure 9:**
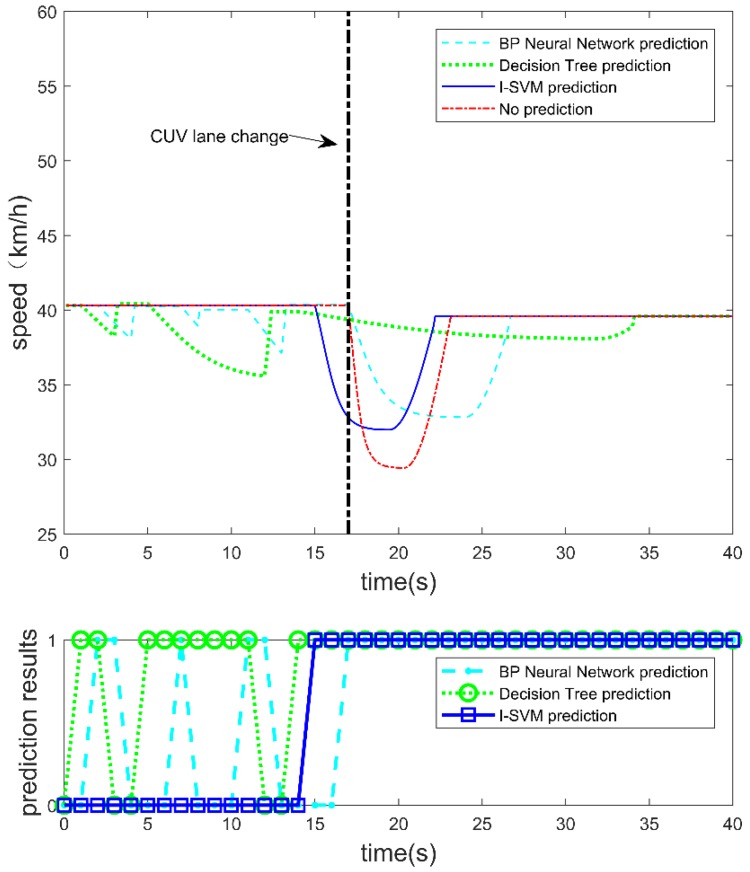
Speed of the autonomous vehicle with interference of CUV lane changing at 17th second.

Figure 9 shows the change of the longitudinal speed of the autonomous vehicle when using the BP neural network, decision tree, and I-SVM for lane change prediction and no lane change prediction. The second chart shows the lane change prediction results of these algorithms. When the prediction result is 1, it means the CUV will make a lane change. When the prediction result is 0, it means no lane change. The first chart shows the speed profile of the autonomous vehicle during the test. I-SVM predicted the lane change behavior at the 15th second and the C-APF controller started to work two seconds before the CUV made a lane change. When the lane change prediction is not carried out, the speed of the autonomous vehicle is reduced from 40.32 km/h to 29.40 km/h. When using the I-SVM to predict the CUV, the speed of the autonomous vehicle is reduced from 40.32 km/h to 31.97 km/h. Compared with no lane change prediction, the speed variant was mitigated. Before the CUV made a lane change, both the BP and decision tree made several wrong predictions, which triggered the safety controller of the autonomous vehicle. 

B. Distance between the autonomous vehicle and the CUV

**Figure 10:**
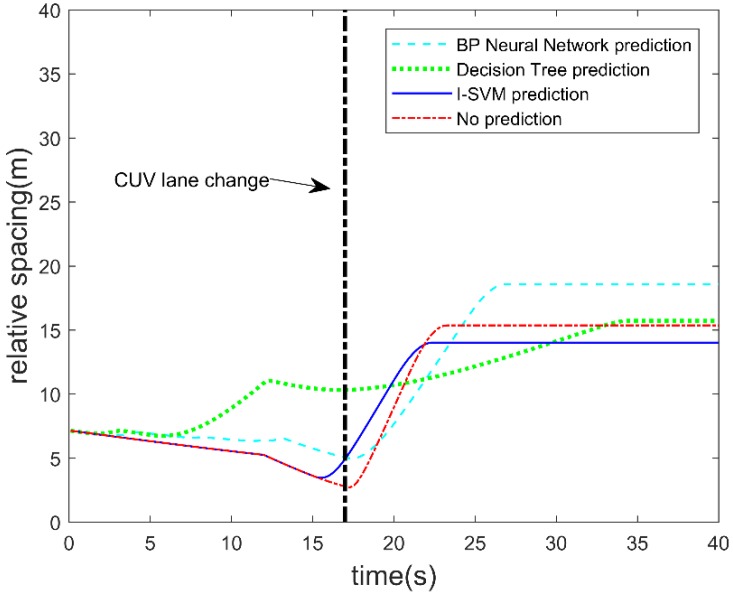
Distance between the autonomous vehicle and the CUV.

Figure 10 shows the variation of the distance between the autonomous vehicle and the CUV. The distance is an intuitive feature for collision. The distance is greater than zero during the whole test, so no collision occurs for each method. At the beginning, the autonomous vehicle’s velocity is greater than that of the CUV, so before these prediction algorithms make a lane change prediction, the distance increases. As soon as a lane change is predicted, the safety controller of the autonomous vehicle will be triggered, and the distance will increase as is shown in the figure. The minimum distance between the autonomous vehicle and the CUV for not lane change prediction is 2.72 m. The minimum distance between the autonomous vehicle and CUV for lane change prediction is 3.46 m. It can be seen that the minimum distance without prediction is bigger than others during the transition process, which increases the risk of collisions. It is clear that the final stable distance converges to different values due to the different strategies taken by the autonomous vehicle during the test. The final stable distance of I-SVM is the shortest among these algorithms, which is conducive to traffic efficiency.

C. Acceleration of the autonomous vehicle

**Figure 11:**
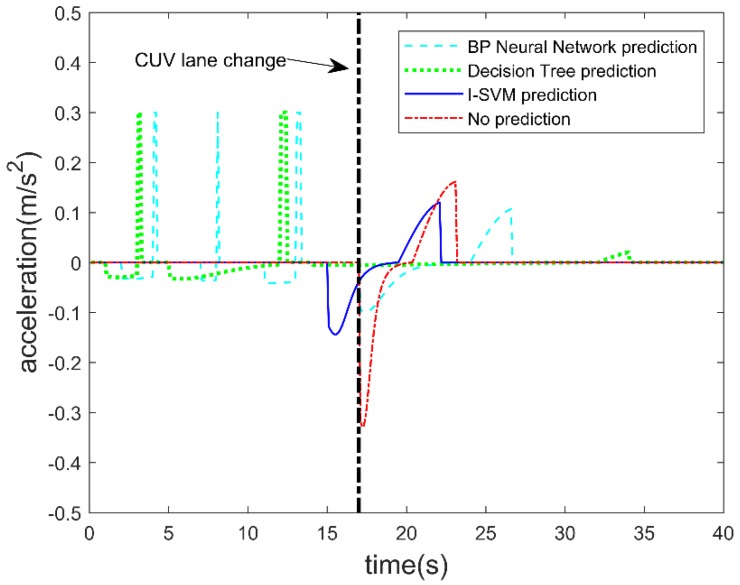
Acceleration of the autonomous vehicle with interference of CUV lane changing at 17th second.

Figure 11 shows the acceleration variation of the autonomous vehicle. If the lane change prediction is not made, the autonomous vehicle needs to be decelerated with a large negative acceleration when the CUV changes lanes. The autonomous vehicle can use a smaller negative deceleration when using the I-SVM algorithm for lane change prediction. It can be seen that the maximum negative acceleration of an autonomous vehicle that performs lane change prediction is –0.13 m/s^2^, and the maximum positive acceleration is 0.12 m/s^2^. The maximum negative acceleration of the autonomous vehicle without the lane change prediction is −0.32 m/s^2^, the maximum positive acceleration is 0.16 m/s^2^. There exists disturbance if a wrong lane change prediction is made, which is obvious from the acceleration variation. Acceleration variation is related to ride comfort and energy consumption, so more moderate acceleration changes are expected for vehicles. Obviously, using the I-SVM to make a lane change prediction for the CUV can make the autonomous vehicle run more smoothly.

D. Energy consumption of the autonomous vehicle 

**Figure 12:**
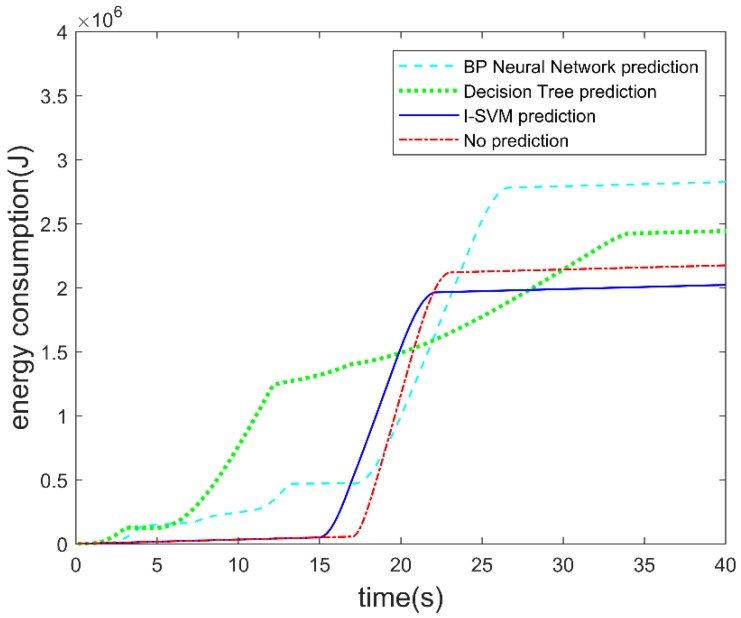
Energy consumption of the autonomous vehicle.

The energy consumption of the autonomous vehicle with different prediction algorithms is shown in Figure 12. The energy consumption of the autonomous vehicle includes loss of kinetic energy, and energy loss due to rolling resistance and windage while the autonomous vehicle is running. The parameters used in the simulation are given in Table 4. Energy consumption is accumulated over time during the test. It can be seen from the figure that prediction of the CUV’s lane change behavior by the I-SVM is more favorable in terms of saving energy consumption. From the simulation result, final energy consumption without lane change prediction is 2.17 × 10^6^ J. Final energy consumption when using BP neural network for lane change prediction is 2.82 × 10^6^ J. Final energy consumption when using decision tree for lane change prediction is 2.44 × 10^6^ J. Final energy consumption when using I-SVM for lane change prediction is 2.02 × 10^6^ J. It shows that using I-SVM for lane change prediction can save 1.5 × 10^5^ J of energy compared to no lane change prediction, and energy consumption is reduced by 6.9%. However, for the BP and decision tree, the total energy consumption is higher; the reason can be seen from Figure 9 and Figure 11. Before the CUV made a lane change, the BP and decision tree made several wrong predictions, which led to deceleration and acceleration during the test scenario. Both deceleration and acceleration will cause energy consumption. Thus, for the autonomous vehicle accurate prediction of the CUV’s lane change behavior is beneficial to energy saving.

E. Jerk of the autonomous vehicle

**Figure 13:**
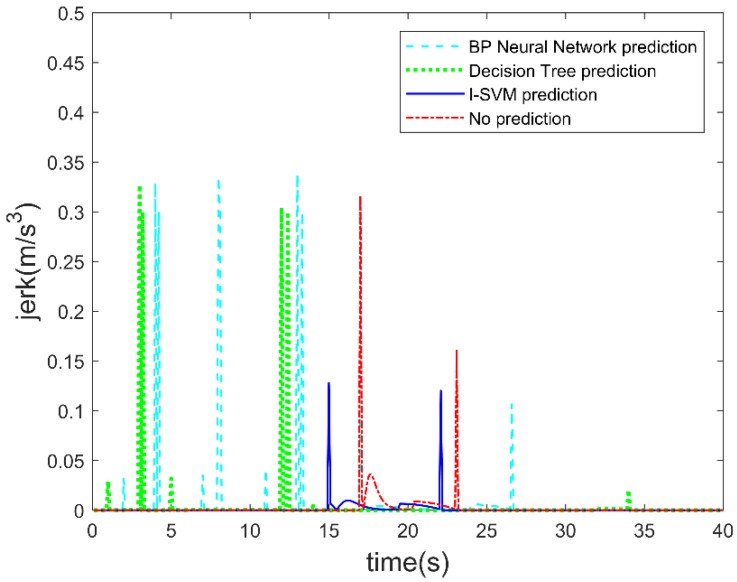
Jerk of the autonomous vehicle.

Figure 13 shows the jerk of the autonomous vehicle during the test with different prediction algorithms. The jerk affects the ride comfort of the autonomous vehicle. Jerk is calculated from the derivative of the acceleration. It can be seen from Figure 13 that when the CUV crosses the lane line, the autonomous vehicle starts to decelerate when the lane change prediction is not used. At this time, the autonomous vehicle and the CUV are closer to each other, so that the repulsive field will generate a larger repulsive force. This causes the autonomous vehicle to decelerate at a greater negative acceleration, which leads to a greater jerk for the autonomous vehicle. When I-SVM is used to predict the CUV’s lane change, the autonomous vehicle can start to decelerate before the lane change. At this time, the repulsive field produces less repulsive force. This allows autonomous vehicles to decelerate with a smaller negative acceleration, which leads to a smaller jerk. The maximum jerk is 0.13 m/s^3^ when the CUV change is predicted by I-SVM, and the maximum jerk is 0.32 m/s^3^ when the CUV lane change is not predicted. The accuracy of the BP neural network and decision tree to predict the lane change is lower than I-SVM and there are several false predictions. This makes the autonomous vehicle frequently accelerate and decelerate, which increases the jerk of the autonomous vehicle. Obviously, predicting the CUV’s lane change behavior by I-SVM can improve the ride comfort of autonomous vehicles. 

## 6. Conclusions and Future work

In this paper, the prediction of CUV lane change intention and safety control of an autonomous vehicle in the adjacent lane was investigated in a mixed traffic environment. An interval-based support vector machine was proposed to predict the CUV’s lane change intention. A general public sample data set was used to train and test the lane change model. The test results show that the prediction accuracy of a 2 s interval before lane change can reach 83.333%. Compared with other complicated lane change prediction methods, the prediction accuracy was reduced by about 10%, at the expense of using less state data. Compared with classical algorithms such as the BP and decision tree, the proposed interval-based support vector machine performs with greater prediction accuracy. A conditional artificial potential field method is used to design the car-following controller by incorporating the lane-change intentions of the CUV, which has low computational complexity and can be used in real-time control for an autonomous vehicle. The simulation results verify its performance from the perspectives of distance, speed, acceleration, energy consumption, and jerk. 

It could be concluded that, by the proposed interval-based support vector machine algorithm, the lane change intention of a connected unintelligent vehicle can be predicted at a favorable accuracy. The prediction was carried out by available sensors on the connected unintelligent vehicles. Lane change prediction would help with safety, energy consumption, and ride comfort of the autonomous vehicle. However, the prediction accuracy would impact the conditional artificial potential field controller response of the autonomous vehicle. These results were shown rigorously in the simulation results conducted in a real concept lane change test scenario of NHTSA (2018).

In the future, more efficient and accurate unintelligent vehicle lane change behavior prediction needs to be studied in depth. The test result shows the prediction accuracy is 83.333%, which may lead to the autonomous vehicle making an unnecessary decision. More efficient and accurate lane change intention prediction needs to be improved with the development of machine learning. Furthermore, the control pattern of autonomous vehicles needs to be more robust to the prediction error.

## Figures and Tables

**Table 1:** Prediction accuracy for different prediction horizons.

Prediction Horizon	Cross-Validation	Training Set	Test Set
18 s	81.875%	100%	83.333%
15 s	59.375%	100%	57.143%
12 s	67.5%	98.125%	52.381%
9 s	66.875%	80%	66.667%
6 s	63.125%	98.125%	59.524%
3 s	65.625%	70%	61.905%

**Table 2:** Prediction accuracy of BP neural network.

Prediction Horizon	Accuracy
18 s	54.55%
15 s	55.12%
12 s	57.64%
9 s	56.91%
6 s	56.83%
3 s	56.59%

**Table 3:** Prediction accuracy of decision tree.

Prediction Horizon	Accuracy
18 s	58.54%
15 s	59.02%
12 s	60.73%
9 s	62.76%
6 s	61.63%
3 s	59.35%

**Table 4:** Simulation parameters.

Parameter Name	Parameter Value
vehicle weight	1650 kg
air resistance coefficient	0.3
windward area of the vehicle	2.05 m^2^
air density	1.2258 N·s^2^·m^−4^
rolling drag coefficient	0.018
